# Mediation effect of body mass index on the association between spicy food intake and hyperuricemia in rural Chinese adults: the Henan rural cohort study

**DOI:** 10.1186/s12889-020-09736-9

**Published:** 2020-10-29

**Authors:** Xiaokang Dong, Yuqian Li, Kaili Yang, Lulu Zhang, Yuan Xue, Songcheng Yu, Xiaotian Liu, Runqi Tu, Dou Qiao, Zhicheng Luo, Xue Liu, Yan Wang, Wenjie Li, Zhaohui Zheng, Chongjian Wang

**Affiliations:** 1 Department of Epidemiology and Biostatistics, College of Public Health, Zhengzhou University, 100 Kexue Avenue, Zhengzhou, 450001 Henan PR China; 2 Department of Clinical Pharmacology, School of Pharmaceutical Science, Zhengzhou University, Henan, Zhengzhou, PR China; 3 Department of Nutrition and Food Hygiene, College of Public Health, Zhengzhou University, Henan, Zhengzhou, PR China; 4 Department of Rheumatology, The First Affiliated Hospital of Zhengzhou University, Henan, Zhengzhou, PR China

**Keywords:** Spicy food intake, BMI, Hyperuricemia, Mediation effect

## Abstract

**Background:**

The relationship of spicy food intake with hyperuricemia remains unknown. The objective of this study was to examine the association between spicy food intake and hyperuricemia, and whether this association was mediated by body mass index (BMI) in Chinese rural population.

**Methods:**

38, 027 adults aged 18–79 years were recruited from the Henan Rural Cohort Study. Information on spicy food intake was obtained using a validated questionnaire survey. Multivariable logistic regression model was used to estimate the association between spicy food intake and hyperuricemia, multiple linear regression model was performed to estimate the relationships between spicy food intake, BMI and serum urate level. BMI was used as a mediator to evaluate the mediation effect.

**Results:**

After adjusting for potential confounders, compared with no spicy food flavor, the odds ratio (*OR*) and 95% confidence interval (*CI*) of mild, middle, and heavy flavor for hyperuricemia were 1.09 (1.00–1.19), 1.10 (0.97–1.24), and 1.21 (1.10–1.46), respectively (*P*_trend_ = 0.017). Similarly, compared with those without intake in spicy food, the multivariable adjusted *OR* (95% *CI*) of 1-2 days/week, 3–5 days/week, and 6–7 days/week were 1.15 (1.01–1.31), 1.14 (1.01–1.30) and 1.15 (1.05–1.26), respectively (*P*_trend_ = 0.007). However, when we further controlling for BMI, the associations were substantially attenuated. Furthermore, mediation analysis showed that BMI play a full mediating role in the relationship of spicy food intake with hyperuricemia.

**Conclusion:**

Spicy food flavor and intake frequency are positively related with hyperuricemia in Chinese rural population. BMI may play a full mediating role in the relationship.

**Trial registration:**

The Henan Rural Cohort Study registered at Chinese Clinical Trial Register (Registration number: ChiCTR-OOC-15006699). Date of registration: 2015-07-06.

## Background

In recent decades, epidemiologic studies show that hyperuricemia has caused many severe public health and social problems all over the world [1, 2]. Hyperuricemia is generally detected in people with abnormal purine metabolism, including overproduction of uric acid (UA) and insufficient UA excretion from the kidneys. According to the Meta-Analysis conducted in China, the prevalence of hyperuricemia between 2000 and 2014 was 13.3% [3]. Recent studies found hyperuricemia was not only associated with an increased risk of developing gout, but also caused chronic kidney disease, cardiovascular diseases and metabolic syndrome [4–6]. In many developed countries, the high incidence of hyperuricemia was correlated with the overweight and obesity, hypertension, hypertriglyceridemia, type 2 diabetes mellitus, hypercholesterolemia, as well as the Western diet rich in purine, alcohol, meat consumption, and soft drinks [7, 8], dietary patterns also play an important role in the development of hyperuricemia.

Spices have been an integral part of culinary cultures around the world and have a long history of use for flavoring, coloring and preserving food, as well as for medicinal purposes [9, 10]. Spiciness or pungency was considered as one of the primary tastes in ancient India and China [11, 12]. There is a large geographic and culture difference in terms of spicy food intake. For example, spicy food intake is higher in Asian than European countries [13]. In China, almost more than 30% of adults consume spicy food daily [14]. Over the past several decades, many studies have explored the effects of spicy food and capsaicin, which is considered as a major bioactive ingredient. Although some previous studies have found the good effects of spicy food intake on obesity and other metabolic diseases [11, 14–16], there are inconsistent findings of the association between spicy food intake and obesity, which were reported in animal and human intervention studies [17, 18]. The China Kadoorie Biobank (CKB) study found that spicy food consumption was associated with elevated body mass index (BMI) and other adiposity measures among a large Chinese population [19]. Our previous study also found spicy flavor, spicy food intake frequency was associated with increased risk of general obesity [20]. Many epidemiologic studies found that obesity was a major risk factor for hyperuricemia [21, 22]. However, the direct association between spicy food intake and hyperuricemia remain unclear and its potential mechanism has not been fully elucidated. Therefore, the current study aimed to investigate the association between spicy food flavor, intake frequency and prevalence of hyperuricemia in the Henan Rural Cohort Study, and to test whether there was a mediating effect on this association by BMI, which is an important obesity index.

## Materials and methods

### Study population

The baseline survey of the Henan Rural Cohort Study was conducted in 5 rural regions of Henan province in China from July 2015 to September 2017. Detailed description on study design and eligibility criteria has been published elsewhere [23, 24]. 39,259 participants aged 18–79 years old were enrolled to complete the questionnaire and physical measurements. The study has been registered in Chinese Clinical Trial Register. To explore the relationship between spicy food intake and hyperuricemia, a total of 1232 subjects were excluded because of no data about SUA levels (*n* = 54); incomplete information of spicy food intake (*n* = 29); cancer and renal disease (*n* = 348); hepatitis and tuberculosis (*n* = 801). Ultimately, the study enrolled 38,027 subjects for the current analysis. Before starting the survey, all participants provided written informed consent, and the protocol was approved by the Zhengzhou University Life Science Ethics Review Committee.

### Assessment of spicy food intake

The validated Food Frequency Questionnaire (FFQ) was used to collected dietary information from individuals [25]. The detailed spicy food information has been described [20]. The average daily spicy food intake including spicy food flavor and intake frequency was estimated using a validated dietary habit questionnaire. Following question was asked: “How much do you like the spicy flavor in your food?” The participants could answer a flavor from four spicy flavors: No, Mild, Middle or Heavy. The participants were also asked “How frequently did you consume spicy foods during a week?” One of following four options could be selected: Never, 1–2 days/week, 3–5 days/ week or 6–7 days/week.

### Ascertainment of hyperuricemia

Venous blood samples were drawn from the subjects who had been fasting overnight. The serum was separated at a rate of 2000 rpm for 10 min, 4 times, and stored at − 20 °C. Serum urate level was measured by ROCHE Cobas C501 automatic biochemical analyzer using enzymatic colorimetric method. In the present study, hyperuricemia was defined as serum urate level > 7.0 mg/dL (417 μmol/L) in men and serum urate level > 6.0 mg/dL (357 μmol/L) in women as the standard definition for some studies [26]. Serum creatinine concentration was measured by the ROCHE Cobas C501 automatic biochemical analyze.

### Assessment and definitions of covariates

The detailed information regarding sociodemographic characteristics (name, sex, age, marital status, educational level), lifestyle factors (smoking status, drinking status, physical activity, dietary habits) and personal histories of chronic diseases (such as type 2 diabetes mellitus, dyslipidemia, and hypertension) were collected by the trained public health investigators using a standardized questionnaire [27]. Education levels was divided into three groups: illiterate or elementary, middle school, and high school or above. Both smoking and drinking status were classified into current, former and never groups. According to the international physical activity questionnaire (IPAQ 2001), physical activity included three categories: low, moderate and high [28]. Data on individual dietary intake were collected by a validated FFQ [25]. The FFQ consisted of 13 main food groups. For everyone, the mean daily total energy intake (including protein, fat and carbohydrate energy intake) was calculated from dietary information according to the Chinese Food Composition Table 2004. The standard principal component analysis method was applied to obtain a four-cluster dietary patterns, dietary pattern I: red meat, white meat and fish; pattern II: vegetables, staple food, and fruits; pattern III: grains, nuts, beans, pickles and animal oils; and pattern IV with milk and eggs [29]. Height and weight were measured by trained physicians, to the nearest 0.1 kg and 0.1 cm separately. Before measurement the subjects need remove their shoes, hats, jackets, overcoats. BMI was calculated as weight divided by height squared (kg/m^2^).

### Statistical analysis

Continuous variables were expressed using means and standard deviations, while percentages were used for categorical variables. Continuous and categorical variables were compared by Analysis of Variance (ANOVA) and chi square tests to determine whether there were differences in covariates among different groups of spicy food intake. To evaluate the linear trend with increasing spicy strength and spicy food frequency, Spearman correlation and Cochran-Mantel-Haenszel tests were used for continuous variables and categorical variables, respectively. Age-standardized prevalence of hyperuricemia was also estimated based on the Population Census 2010 across different groups of spicy food intake.

Multivariable-adjusted logistic regression models were employed to evaluate the association between spicy food flavor, intake frequency and hyperuricemia, and the values of odd ratios (*ORs*) and 95% confidence intervals (*CIs*) were calculated. A set of models were performed to minimize the effect of confounders on this association. Model 1 was crude model, and model 2 adjusted for age, gender, education level, marital status, smoking and drinking status, physical activity, individual dietary pattern, total energy, serum creatinine and model 3 additionally adjusted for type 2 diabetes mellitus (T2DM), hypertension and dyslipidemia status, and model 4 further adjusted for BMI. Multiple linear regression analyses were further performed to explore the relationships between spicy food intake, BMI and serum urate level, with three models.

In order to further explore whether BMI played a mediate role in the association of spicy food intake with hyperuricemia, a mediation analysis was applied. The mediator needed to be continuous variable as previously described elsewhere [30, 31]. Same confounders in model 3 were also adjusted in the mediation analysis. Several main paths were included in the mediation analysis. Path a: the association between spicy food intake and BMI (the mediator); Path b: the association of BMI with hyperuricemia (outcome); Path c and Path c’: the total and direct effects of spicy food intake on hyperuricemia, respectively. Full mediation effect exists when the indirect effect is significant but not for the direct effect. All statistical analyses were conducted by using SPSS version 21.0 and STATA version 13.1. All tests were two sided and *P* values<0.05 indicated statistical significance.

## Results

### General characteristics

The median age of 38,027 subjects was 55.53 ± 12.21 years in the total population. Table 1 presents the general characteristics of the participants by categories of the spicy food flavor and the spicy food intake frequency. Participants with heavier spicy flavor were more likely to be younger in age, be male with married or cohabiting, have more total energy intake, less likely to be drinker and smoker, more physical activity, higher BMI and serum urate level, and less T2DM and hypertension status (all *P*_trend_ < 0.001). Similar differences of distribution in these selected variables were also found among four groups of intake frequency (all *P*_trend_ < 0.001).

**Table 1:** Sociodemographic characteristics of participants grouped by spicy food flavor and intake frequency

Variable	Spicy food flavor								***P***_**trend**_	Spicy food intake frequency								***P***_**trend**_
	No		Mild		Middle		Heavy			Never		1-2d/week		3-5d/week		6–7 d/week		
	Mean	SD	Mean	SD	Mean	SD	Mean	SD		Mean	SD	Mean	SD	Mean	SD	Mean	SD	
No. participants	16,282		14,889		5326		1530			12,361		3268		3264		10,026		
Age (years)	58.3	11.4	53.9	12.3	52.3	12.4	52.1	11.5	< 0.001	58.2	11.7	51.6	13.2	51.4	13.5	54.4	11.6	< 0.001
Male (%)	37.8		38.4		44.1		46.4		< 0.001	39.1		38.1		44.6		42.1		< 0.001
Educational level (%)									< 0.001									< 0.001
Illiterate and Elementary	49.0		41.5		41.0		42.8			48.4		33.6		34.7		45.6		
Middle school	37.1		41.8		41.9		43.5			36.1		41.4		41.4		40.8		
High school and above	13.9		16.7		17.2		13.7			15.5		25.0		23.9		13.7		
Marital status (%)									< 0.001									< 0.001
Married/cohabiting	87.9		90.7		91.9		92.0			88.2		91.4		90.8		91.7		
Widowed/single/divorced/separation	12.1		9.3		8.1		8.0			11.8		8.6		9.2		8.3		
Smoking status(%)									< 0.001									< 0.001
Nonsmoker	76.6		72.4		66.5		61.1			75.6		74.6		67.0		68.3		
Ex-smoker	8.6		7.7		7.1		6.8			8.4		6.5		7.9		7.4		
smoker	14.9		19.9		26.4		32.1			16.0		18.8		25.2		24.3		
Drinking status(%)									< 0.001									< 0.001
Nondrinker	82.7		75.6		68.1		68.9			83.2		77.2		72.1		72.6		
Ex-drinker	5.3		4.0		4.1		4.4			5.3		3.7		4.4		4.8		
Drinker	12.1		20.4		27.7		26.7			11.5		19.0		23.5		22.6		
Physical activity (%)									< 0.001									< 0.001
Low	34.8		30.8		28.7		31.4			35.8		34.4		31.8		26.1		
Middle	37.2		38.9		37.2		35.9			35.4		32.3		37.5		39.7		
High	28.0		30.3		34.0		32.7			28.8		33.3		30.8		34.2		
Dietary pattern									< 0.001									< 0.001
Pattern I	18.6		20.9		23.2		27.5			18.7		20.9		22.6		21.8		
Pattern II	30.7		35.3		33.9		38.8			28.6		29.1		30.2		41.3		
Pattern III	22.4		19.2		20.1		16.4			23.0		22.3		20.8		16.9		
Pattern IV	28.4		24.6		22.9		17.3			29.7		27.6		26.4		20.0		
Total energy intake (kcal/d)	2387	661.1	2467	663.9	2531	691.2	2700	695.3	< 0.001	2368	662.8	2367	652.6	2443	662.1	2604	693.1	< 0.001
BMI (kg/m ^2^)	24.63	3.52	24.94	3.51	25.20	3.75	25.13	3.68	< 0.001	24.57	3.51	25.01	3.62	24.83	3.66	24.85	3.57	< 0.001
Serum creatinine (umol /L)	62.58	14.9	61.58	13.72	61.45	13.37	62.68	12.94	< 0.001	63.23	15.27	62.52	14.74	63.78	14.41	62.36	13.07	< 0.001
Serum urate level (umol /L)	282.43	77.0	287.7	80.5	290.7	82.8	301.6	84.1	< 0.001	288.5	77.6	296.9	83.9	301.4	86.7	297.2	81.2	< 0.001
T2DM (%)	10.6		8.5		8.88		8.6		< 0.001	10.0		8.1		7.4		8.0		< 0.001
Hypertension (%)	36.5		30.88		29.5		22.7		< 0.001	36.2		31.7		30.2		27.7		< 0.001
Dyslipidemia (%)	37.8		37.0		38.4		39.5		0.084	38.7		38.8		38.4		38.7		0.984

*SD* Standard deviation, *T2DM* Type 2 diabetes mellitus

### Prevalence of hyperuricemia

**Fig. 1:**
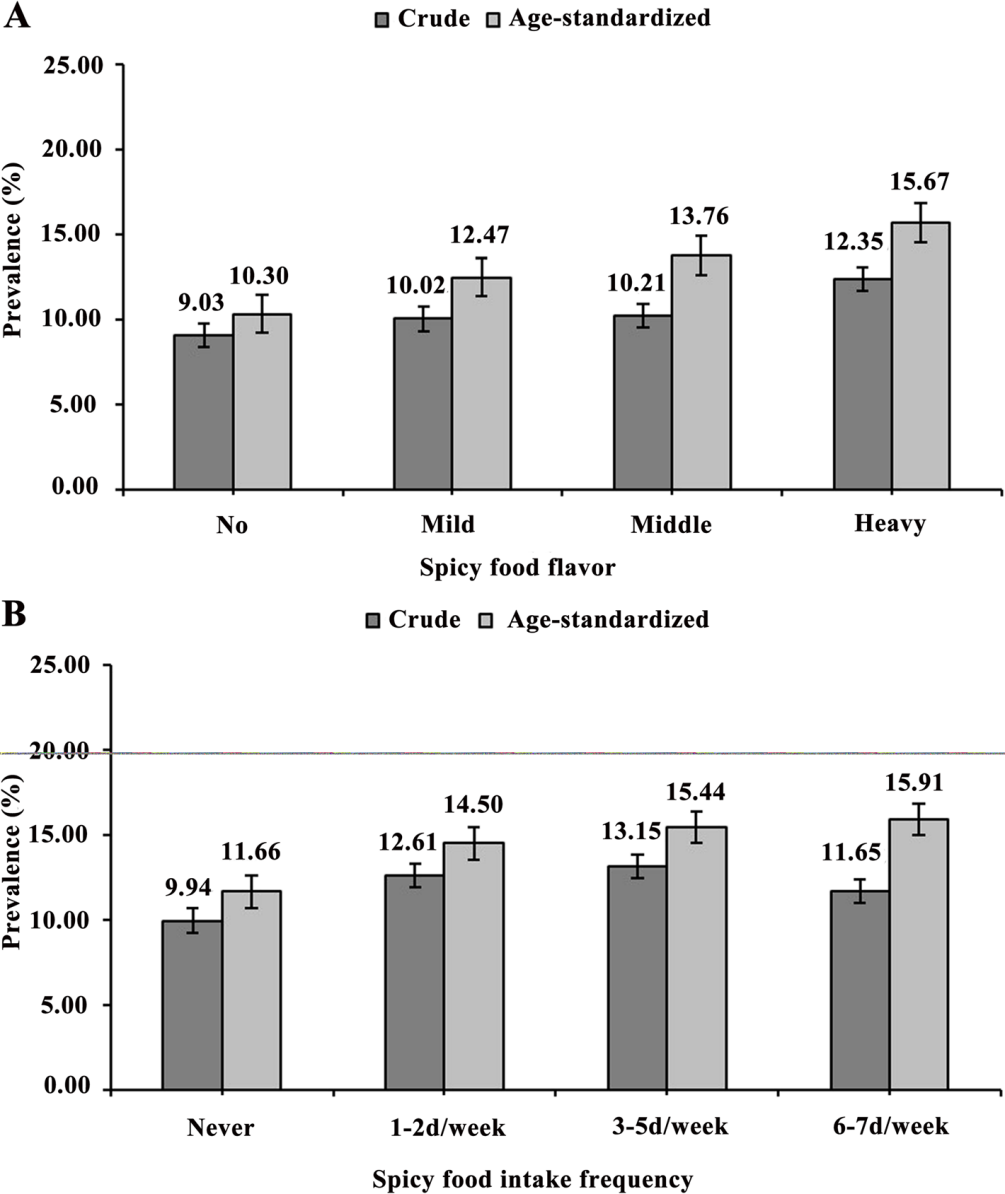
The crude and standardized prevalence of hyperuricemia according to spicy food flavor (**a**) and spicy food intake frequency (**b**). Black bars indicate 95% *CI*

The prevalence of hyperuricemia across different categories of spicy food flavor and intake frequency is displayed in Fig. 1. In all participants, the crude prevalence of hyperuricemia with No, Mild, Middle, and Heavy spicy flavors were 9.03, 10.02, 10.21 and 11.90%, respectively; and the corresponding age-standardized hyperuricemia prevalence were 10.30, 12.47, 13.76 and 15.67%, respectively (Fig. 1a). Similarly, the crude prevalence of hyperuricemia with intake frequency of Never, 1–2 d/week, 3–5 d/ week and 6–7 d/week were 9.94, 12.61, 13.15, and 11.65%, respectively; and the corresponding age-standardized hyperuricemia prevalence were 11.66, 14.50, 15.44, and 15.91%, respectively (Fig. 1b). In addition, increased trends in the prevalence of hyperuricemia were observed with the increasing level of spicy flavor and intake frequency (all *P*_trend_ < 0.001).

### Association of spicy food intake with hyperuricemia

As shown in Table 2, the findings revealed a positive relationship between spicy food intake and hyperuricemia. After controlling for multiple variables in model 3, the *OR*s (95% *CI*s) of Mild, Middle, and Heavy flavors compared with no spicy food flavor were 1.09 (1.00–1.19), 1.10 (0.97–1.24) and 1.21 (1.10–1.46), respectively (*P*_trend_ = 0.017). The adjusted *OR* (95% *CI*) for each level increment in spicy flavor strength was 1.06 (1.01–1.10). Compared with those without intake in spicy food, participants in intake frequency of 1–2 days/week, 3–5 days/week, and 6–7 days/week were 1.15, 1.14 and 1.15 times more likely to have hyperuricemia (*P*_trend_ = 0.007). The adjusted *OR* (95% *CI*) for each level increment in spicy food intake frequency was 1.02 (1.01–1.03). But the association of spicy food flavor or intake frequency with hyperuricemia was significantly attenuated after additional adjustment for BMI in model 4 (all *P*_trend_ > 0.05).

**Table 2:** *OR* (95%*CI*) of hyperuricemia grouped by spicy food flavor and intake frequency

	Events/N	Model 1	Model 2	Model 3	Model 4
**Spicy food flavor**		**OR (95%*CI*)**	**OR (95%*CI*)**	**OR (95%*CI*)**	**OR (95%*CI*)**
**No**	1366/16282	1.00 (Reference)	1.00 (Reference)	1.00 (Reference)	1.00 (Reference)
**Mild**	1750/14889	1.12 (1.04, 1.21)	1.10 (1.00, 1.20)	1.09 (1.00, 1.19)	1.05 (0.96, 1.15)
**Middle**	611/5326	1.15 (1.03, 1.27)	1.11 (0.99, 1.26)	1.10 (0.97, 1.24)	1.02 (0.91, 1.16)
**Heavy**	181/1530	1.42 (1.21, 1.67)	1.21 (1.01, 1.45)	1.21(1.10, 1.46)	1.13 (0.93, 1.36)
**Each level increment**		1.10 (1.06, 1.14)	1.06 (1.01, 1.11)	1.06 (1.01, 1.10)	1.03 (0.98, 1.08)
***P***_**trend**_		< 0.001	0.011	0.017	0.276
**Spicy food intake frequency**					
**Never**	762/12361	1.00 (Reference)	1.00 (Reference)	1.00 (Reference)	1.00 (Reference)
**1-2d/week**	265/3268	1.31 (1.16, 1.47)	1.18 (1.04, 1.34)	1.15 (1.01, 1.31)	1.10 (0.97, 1.25)
**3-5d/week**	511/3246	1.37 (1.22, 1.54)	1.16 (1.03, 1.32)	1.14 (1.01, 1.30)	1.10 (0.97, 1.26)
**6-7d/week**	1180/10026	1.19 (1.10, 1.30)	1.15 (1.05, 1.26)	1.15 (1.05, 1.26)	1.10 (0.99, 1.21)
**Each level increment**		1.02 (1.01, 1.04)	1.02 (1.01, 1.03)	1.02 (1.01, 1.03)	1.01 (0.99, 1.02)
***P***_**trend**_		< 0.001	0.007	0.007	0.078

Model 1: unadjusted

Model 2: adjusted for age, gender, education level, marital status, smoking and drinking status, physical activity, dietary pattern, serum creatinine, total energy intake

Model 3: adjusted for model 2 plus T2DM, hypertension and dyslipidemia status

Model 4: adjusted for model 2 plus BMI

### Association of spicy food intake with BMI and serum urate level

The associations of spicy food flavor, intake frequency with BMI and serum urate level are presented in Table 3. After adjusting for potential confounders in model 2, compared with no spicy food flavor, the *β* Coefficients and 95% *CI* of Mild, Middle, and Heavy with BMI were 0.29 (0.20, 0.38), 0.50 (0.37, 0.63) and 0.49 (0.28, 0.70), respectively (*P*_trend_ < 0.001). Compared with those without intake in spicy food, the adjusted *ORs* (95% *CIs*) for 1–2 days/week, 3–5 days/week, and 6–7 days/week were 0.39 (0.26, 0.53), 0.26 (0.12, 0.40) and 0.27 (0.17, 0.36), respectively. Similarly, in model 3, the Mild, Middle, and Heavy flavor were associated with a 5.27 μmol/L (95% *CI*: 3.47, 7.08), 4.62 μmol/L (2.08, 7.17) and 10.78 μmol/L (6.70, 14.86) higher serum urate levels; the 1–2 days/week, 3–5 days/week, and 6–7 days/week intake frequency were associated with 5.29 μmol/L (95% *CI*: 2.59, 7.12), 4.40 μmol/L (1.69, 7.12) and 5.80 μmol/L (3.91, 7.68) higher serum urate levels.

**Table 3:** The association (*β* coefficients and *95% CI*) of spicy food flavor or intake frequency with BMI and serum urate level

	Spicy food flavor				Spicy food intake frequency			
	No (***n*** = 16,282)	Mild (***n*** = 14,889)	Middle (***n*** = 5326)	Heavy (***n*** = 1530)	Never (***n*** = 12,361)	1-2d/week (***n*** = 3268)	3-5d/week (***n*** = 3246)	6-7d/week (***n*** = 10,026)
**BMI (kg/m** ^**2**^**)**								
**Model 1**	Reference	0.30 (0.22, 0.38)	0.56 (0.45, 0.67)	0.51 (0.33, 0.70)	Reference	0.44 (0.30, 0.57)	0.26 (0.13, 0.40)	0.28 (0.19, 0.38)
**Model 2**	Reference	0.29 (0.20, 0.38)	0.50 (0.37, 0.63)	0.49 (0.28, 0.70)	Reference	0.39 (0.26, 0.53)	0.26 (0.12, 0.40)	0.27 (0.17 0.36)
**Serum urate level (**μmol**/L)**								
**Model 1**	Reference	5.32 (3.55, 7.08)	8.24 (5.78, 10.70)	19.13 (14.96, 23.30)	Reference	8.35 (5.24, 11.46)	12.79 (9.67, 15.91)	8.67 (6.54, 10.79)
**Model 3**	Reference	5.27 (3.47, 7.08)	4.62 (2.08, 7.17)	10.78 (6.70, 14.86)	Reference	5.29 (2.59, 7.99)	4.40 (1.69, 7.12)	5.80 (3.91, 7.68)

Model 1: unadjusted

Model 2: adjusted for age, gender, education level, marital status, smoking and drinking status, physical activity, dietary pattern, total energy intake

Model 3: adjusted for age, gender, education level, marital status, smoking and drinking status, physical activity, dietary pattern, total energy intake, serum creatinine

### Mediating role of BMI

**Fig. 2:**
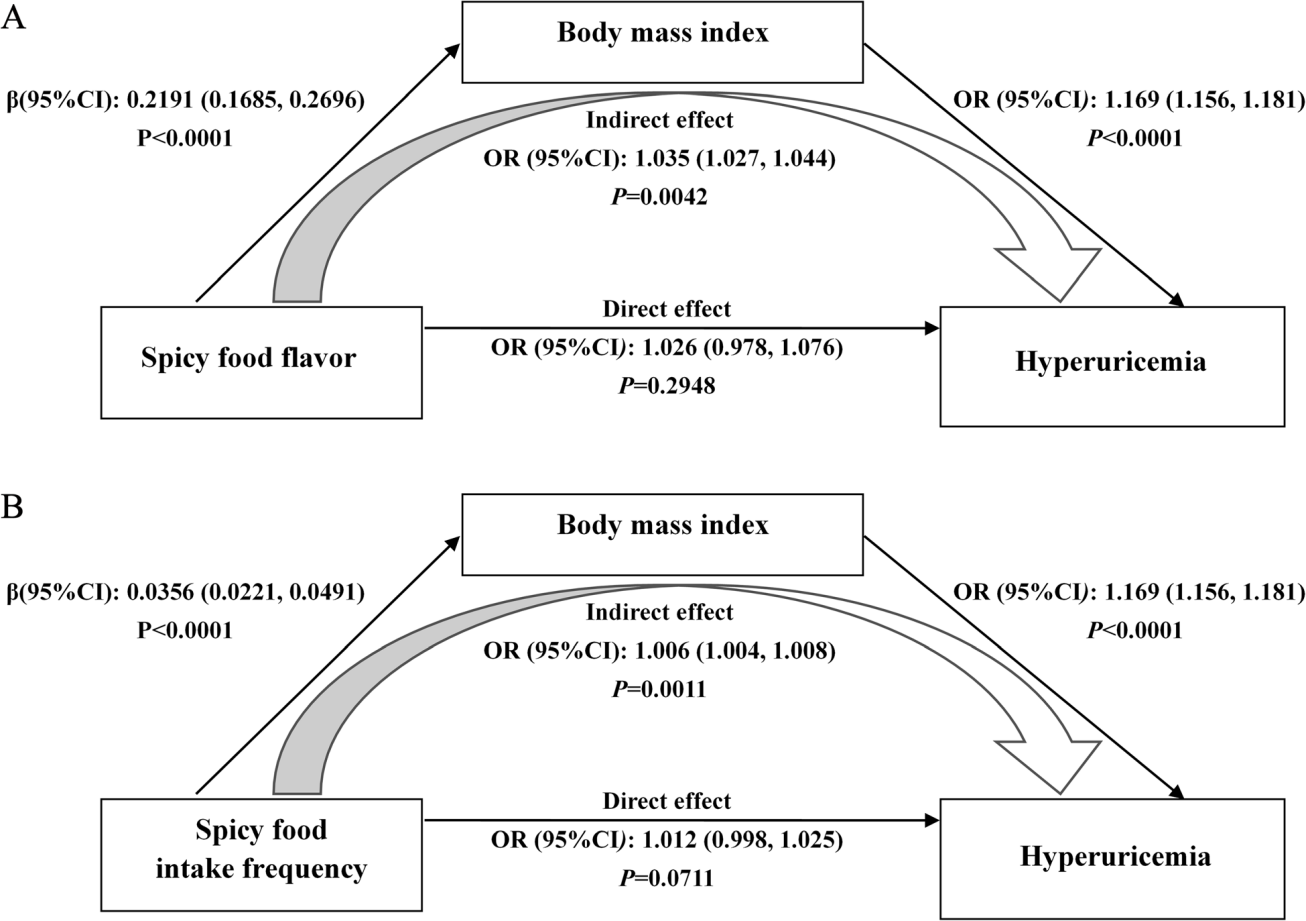
Mediation effect to BMI on the relationship between spicy food flavor (**a**) or intake frequency (**b**) and hyperuricemia. Adjusted for age, gender, education level, smoking and drinking status, alcohol use, physical activity, dietary pattern, total energy intake, serum creatinine, T2DM, hypertension and dyslipidemia status. BMI, body mass index; *CI*, confidence interval

Table 4 provides results of mediation analyses and Fig. 2 demonstrates the model process of BMI mediated the relationship of spicy food intake with hyperuricemia. The total effect spicy food flavor on hyperuricemia was significant (total effect, *OR* = 1.062; 95% *CI* = 1.014–1.112; *P* = 0.011). The estimated *ORs* (95% *CIs* and *P* value) of a significant indirect effect mediated by BMI and a nonsignificant direct effect were 1.035 (95% *CI* = 1.027–1.044 and *P* = 0.0042) and 1.026 (95% *CI* = 0.978–1.076 and *P* = 0.2948), respectively (Fig. 2a). Similarly, the total effect spicy food flavor on hyperuricemia was also significant (total effect, *OR* = 1.018; 95% *CI* = 1.005–1.030; *P* = 0.007). A significant indirect effect mediated by BMI (*OR* = 1.006; 95% *CI* = 1.004–1.008; *P* = 0.0011) and a nonsignificant direct effect (*OR* = 1.012; 95% *CI* = 0.998–1.025; *P* = 0.0711) were also included in the mediating process (Fig. 2b). The results verified our hypothesis that the association of spicy food intake with hyperuricemia was mediated by BMI.

**Table 4:** Mediation analysis of the relationship between spicy food flavor or intake frequency and hyperuricemia by BMI

	Spicy food flavor		Spicy food intake frequency	
Mediation analysis	Parameter estimate (95% ***CI***)	OR (95% ***CI***)	Parameter estimate (95% ***CI***)	***OR*** (95% ***CI***)
Total effect	0.0598 (0.0136, 0.1060)	1.062 (1.014, 1.112)	0.0175 (0.0048, 0.0301)	1.018 (1.005, 1.030)
Direct effect path c’	0.0255 (−0.0222, 0.0731)	1.026 (0.978, 1.076)	0.0120 (−0.0010, 0.0250)	1.012 (0.998, 1.025)
Path a	0.2191 (0.1685, 0.2696)	1.245 (1.184, 1.309)	0.0356 (0.0221, 0.0491)	1.036 (1.022, 1.050)
Path b	0.1560 (0.1454, 0.1667)	1.169 (1.156, 1.181)	0.1560 (0.1454, 0.1667)	1.169 (1.156, 1.181)
Indirect effect path ab	0.0342 (0.0266, 0.0426)	1.035 (1.027, 1.044)	0.0055 (0.0035, 0.0079)	1.006 (1.004, 1.008)

Adjusted for age, gender, education level, marital status, smoking and drinking status, physical activity, dietary pattern, total energy intake, serum creatinine, T2DM, hypertension and dyslipidemia status

Path c’ indicates the path from spicy food flavor or intake frequency to hyperuricemia (Outcome) when controlled for BMI (Mediator). Path a indicates the path from spicy food flavor or intake frequency to BMI (Mediator), Path b indicates the path from BMI (mediator) to hyperuricemia (Outcome). Path ab coefficients represent 5000 bootstrapped samples and bias-corrected 95% *CI*s

*BMI* Body mass index, *OR* Odd ratio, *CI* Confidence interval, *T2DM* Type 2 diabetes mellitus

## Discussion

This study is the first study to explore the relationship between spicy food intake and hyperuricemia among rural Chinese adults. We found that both spicy food flavor and intake frequency were positively associated with the prevalence of hyperuricemia. Furthermore, a higher level of spicy food flavor or intake frequency tended to be associated with a higher BMI and serum urate level. In addition, the relationship of spicy food intake with hyperuricemia was mediated by BMI.

The current study revealed that both spicy food flavor and spicy food intake frequency were positively associated with BMI, serum urate level and risk of hyperuricemia. Although it is the first time using cross-sectional study to find the relationship of spicy food intake with hyperuricemia, there are many previous studies which can support the current results. Firstly, a large amount of studies had proved that obesity is an independent influencing factor for hyperuricemia in US [21], China [32], Japan [33], and Korean patients [34] despite the difference in race, cross-sectional, case-control, or cohort study design. In addition, some large sample studies had found the association of spicy food consumption with obesity, as our previous study have found that spicy flavor and intake frequency were associated with increased risk of general or abdominal obesity [20], it is consistent with the findings of CKB, which reported that the spicy food strength and frequency might associate with elevated BMI and other obesity measures [19]. Moreover, a recent cross-sectional study also showed that spicy food intake can increase the risk of overweight/obesity [35]. Secondly, some studies found the association between spicy food consumption and abnormal lipid metabolism in adults [36] and older people in China [37], respectively. Abnormal lipid metabolism can cause dyslipidemia, as an important risk factor for hyperuricemia was observed in series cross-sectional studies and longitudinal studies [21, 32, 34]. In addition, a study in Korean found that a strong preference for spicy food may be a risk factor for alcohol dependence [38]. Alcohol intake can promote the synthesis of uric acid and subsequently hinder the excretion of uric acid, which finally lead to hyperuricemia [33, 39]. Therefore, the indirect association between spicy food intake and hyperuricemia from both cross-sectional and longitudinal analyses largely conclude the possibility of this positive causation.

In the present study, the association between spicy food intake and hyperuricemia still remained robust after controlling for multiple potential confounding factors in model 3 including drinking status, dyslipidemia status, dietary pattern, total energy intake and so on. But when we further controlled for BMI, the association became nonsignificant. Furthermore, in the mediation analysis BMI played a full mediation with spicy food flavor or intake frequency and hyperuricemia. These findings supported our hypothesis, it seems plausible that spicy food intake predisposes to high BMI and general obesity [19, 20], and in turn to high serum uric acid and hyperuricemia [32].

Although the exact mechanisms of the adverse effects of spicy food intake on hyperuricemia are yet to be elucidated, several potential hypotheses have been proposed. Firstly, a underlying reason for the association may be explained by the meat-based diet with chili intake in Chinese cuisines [40]. In Chinese cuisines, spicy food is inclined to be more meat-based rather than vegetable-based, excessive fat meat intake with spicy foods may increase the risk of obesity [19], general obesity is an independent risk factor for hyperuricemia. Spicy food intake also may increase carbohydrates intake to relieve the burning sensation [20], which might lead to weight increase and a high BMI. Fat accumulation may cause excessive uric acid production, which in turn result in an elevated influx of plasma free fatty acid into the portal vein and liver, stimulation of neutral fat synthesis, and a consequent attendant surge in uric acid production in the activated uric acid synthesis pathway [33]. Moreover, compared with other foods, meat contains more purines. In China, spicy flavor is often used for flavor of mutton, fish, particularly hotpot that contains plenty of purine [19, 41]. The excessive purines intake with spicy foods can directly cause high serum uric acid. As a consequence, it could further develop into hyperuricemia [41]. Secondly, in China diet, chili sauce and chili oil are widely used for flavoring [24], which may increase fat intake, and further elevate serum lipid levels. The increase of lipid levels especially the triglycerides (TG) will induce more free fatty acid production, accelerate the decomposition of adenosine triphosphate, and increase the production of uric acid [42]. In our study, although many possible confounders such as demographics, lifestyle, dietary pattern, total energy intake and personal disease status have been adjusted, the positive association between spicy food intake and hyperuricemia remained virtually unchanged. In addition, some unmeasured factors like accurate chilli and purine intake could not be fully controlled. Thus further clinical research and prospective studies in general population are essential to elucidate the mechanisms of the relationship spicy food intake with hyperuricemia.

This study was the first observational study to explore whether the BMI as mediation role on spicy food intake and hyperuricemia. The current study also includes a large sample rural population and adjustment for many established and potential risk factors for hyperuricemia, which provide sufficient statistical power. However, several limitations of the study should be acknowledged. Firstly, the study was conducted based on design of cross-sectional study, which may not be able to accurately determine the cause-effect association. Secondly, because the assessment of spicy food intake and other dietary information of participants were collected through FFQ, the recall bias cannot be neglected. However, a validation study had been conducted with the 3-day 24 h recall to confirm that the FFQ is a representative tool to obtain reliable dietary data on a rural population [25]. Thirdly, although wide range risk factors for hyperuricemia were controlled, the lack of some other unmeasured and unselected covariates (such as the actual data of chili and purine intake) were not considered, which may be limited our ability to explore the mechanism of spicy food on hyperuricemia. Finally, our participants are from Henan rural areas in China, which may be limited to expand these findings to other countries and areas. Further validation of the current results in other countries and areas is necessary in the later study. On all accounts, the findings based on the large sample population study can provide us with some prospective about the association of spicy food intake with hyperuricemia in Chinese rural population.

## Conclusion

The current analysis showed significant positive association of spicy food flavor, spicy food intake frequency with hyperuricemia prevalence. Furthermore, BMI may play a mediator effect in this association. The positive associations of spicy food intake with BMI and prevalence of hyperuricemia suggest that we not only need to care about the food we intake, but also pay attention to the spicy itself. Reducing spicy food intake (both spicy flavor and intake frequency) may be a beneficial way to improve metabolic health.

## Data Availability

The data used in this study are available. It will be shared on reasonable request to the corresponding author.

## Abbreviations

BMI: Body mass index
T2DM: Type 2 diabetes mellitus
*OR*: Odds ratio
*CI*: Confidence interval
CVD: Cardiovascular diseases
ANOVA: Analysis of Variance
FFQ: Food Frequency Questionnaire
CKB: China Kadoorie Biobank
*SD*: Standard deviation
TG: Triglycerides
